# Are There Better Methods of Monitoring MRSA Control than Bacteraemia Surveillance? An Observational Database Study

**DOI:** 10.1371/journal.pone.0002378

**Published:** 2008-06-11

**Authors:** Sarah Walker, Tim E. A. Peto, Lily O'Connor, Derrick W. Crook, David Wyllie

**Affiliations:** 1 Oxford Radcliffe Hospitals NHS Trust, John Radcliffe Hospital, Oxford, United Kingdom; 2 MRC Clinical Trials Unit, London, United Kingdom; 3 Nuffield Department of Clinical Laboratory Sciences, University of Oxford, Oxford, United Kingdom; Brown University School of Medicine, United States of America

## Abstract

**Background:**

Despite a substantial burden of non-bacteraemic methicillin resistant *Staphylococcus aureus* (MRSA) disease, most MRSA surveillance schemes are based on bacteraemias. Using bacteraemia as an outcome, trends at hospital level are difficult to discern, due to random variation. We investigated rates of nosocomial bacteraemic and non-bacteraemic MRSA infection as surveillance outcomes.

**Methods and Findings:**

We used microbiology and patient administration system data from an Oxford hospital to estimate monthly rates of first nosocomial MRSA bacteraemia, and nosocomial MRSA isolation from blood/respiratory/sterile site specimens (“sterile sites”) or all clinical samples (screens excluded) in all patients admitted from the community for at least 2 days between April 1998 and June 2006. During this period there were 441 nosocomial MRSA bacteraemias, 1464 MRSA isolations from sterile sites, and 3450 isolations from clinical specimens (8% blood, 15% sterile site, 10% respiratory, 59% surface swabs, 8% urine) in over 2.6 million patient-days. The ratio of bacteraemias to sterile site and all clinical isolations was similar over this period (around 3 and 8-fold lower respectively), during which rates of nosocomial MRSA bacteraemia increased by 27% per year to July 2003 before decreasing by 18% per year thereafter (heterogeneity p<0.001). Trends in sterile site and all clinical isolations were similar. Notably, a change in rate of all clinical MRSA isolations in December 2002 could first be detected with conventional statistical significance by August 2003 (p = 0.03). In contrast, when monitoring MRSA bacteraemia, identification of probable changes in trend took longer, first achieving p<0.05 in July 2004.

**Conclusions:**

MRSA isolation from all sites of suspected infection, including bacteraemic and non-bacteraemic isolation, is a potential new surveillance method for MRSA control. It occurs about 8 times more frequently than bacteraemia, allowing robust statistical determination of changing rates over substantially shorter times or smaller areas than using bacteraemia as an outcome.

## Introduction

*Staphylococcus aureus* is responsible for a substantial burden of nosocomial disease; for example, being named in 8% of hospital discharge diagnoses in a recent study in the United States[1]. In the UK, which has had a large outbreak of two clones of methicillin-resistant *S. aureus* (MRSA) for the last fifteen years, MRSA now accounts for 37% of *S. aureus* blood stream isolates[2], with evidence that it has added to the burden of disease caused by methicillin sensitive *S. aureus* [3]. Consequently, reduction in MRSA disease burden has become a key UK government priority, with a much publicised target of a 50% reduction in MRSA bacteraemia by 2008. Despite this, there have been very few rigorous evaluations of how best to control MRSA[4], with optimal metrics for examining the effect of interventions little studied. MRSA bacteraemia is a serious outcome, amendable to passive surveillance; however, it is recognised that monitoring using bacteraemia rates necessitates long periods of follow-up to determine whether infection control interventions have had an effect in a hospital or hospital subunit[5].

Nosocomial isolation of MRSA from sites other than blood is also of clinical significance, and can arise in two settings: one in which the patient is clinically infected, and the other in which they are not and are carriers of the organism. Carriage of *Staphylococcus aureus* is a well-recognised precedent of infection with the same strain[6], and multiple studies show MRSA isolation from diverse sites, including ulcers, is associated with high risk of subsequent clinical infection[7]–[9]. Isolation of *S. aureus* is common with ventilator associated pneumonia[10] and surgical site infections[11]: for both, MRSA increases morbidity, hospital stay and costs relative to methicillin sensitive strains[12]–[15].

Here, we describe nosocomial MRSA bacteraemia and isolations from other clinical samples in a large teaching hospital over a 10 year period, demonstrating that changes over time are very similar, but that non-bacteraemic isolations are about 8 times more common. We further investigate whether, using MRSA isolation from all sites of suspected infection as a surveillance measure, more rapid and precise estimates of trends in nosocomial MRSA isolation can be formed than by the use of bacteraemic isolates alone.

## Methods

### Participants

Our study included data from the John Radcliffe Hospital, the Radcliffe Infirmary, the Churchill Hospital and the Horton General Hospital (the Oxford Radcliffe Trust, ORH, UK), which offer the majority of specialist regional services plus acute clinical and bacteriology services to about 600,000 people. Admissions to other much smaller hospitals in the area (including a specialist orthopaedic hospital, psychiatric hospitals, and several community hospitals) were not included. Microbiological processing used standard techniques, as recommended by the standard operating procedures of the Health Protection Agency. Patient admissions, excluding outpatients, between 1 January 1997 (1 January 1999 for Horton) and 31 July 2006 were anonymously linked to information on microbiology isolates from 1 January 1995 and 31 July 2006, using previously described methods[3]. Here, we restrict analysis to 1 April 1998 to 30 June 2006 to ensure that exposure to MRSA before admission can be estimated for at least 3 years in all cases, and discharge status is known on almost all the cohort.

### Outcomes

The outcomes considered were

- nosocomial isolations of MRSA[3], defined as isolations from patients admitted to ORH for at least the previous 2 days from
  - blood cultures
  - blood cultures and all other samples taken due to clinical suspicion of infection (non-screening “clinical samples”), except for vascular line tip cultures, which we excluded because of their relationship to blood cultures.
  - blood cultures and clinical samples only from respiratory and normally sterile sites taken under aseptic technique, such ascites, joint aspirates, cerebrospinal fluid, pre-prosthetic material, and collections of pus.

Following the principle that an outcome should be counted if either the event of interest or a more serious event has occurred, we compared MRSA isolation from blood cultures with isolation from both blood cultures and other specimens (rather than other specimens alone), analogous to, for example, comparing time to death and time to myocardial infarction or death.

For blood, blood/respiratory/sterile site and all clinical isolates, we analysed those samples:

- taken during the period 1 April 1998 and 30 June 2006
- when there was no positive MRSA isolation from that group of sites within the previous 14 days, analogous to the process recommended for bacteraemia reporting[2]
- which were the first such positive isolation per admission, to focus on new infections rather than repeat isolations which could be influenced by persistent, unresolved infected sites (such as fistulae and wound drains).

We excluded the following admissions from analysis:

- those with MRSA isolated from the group of sites within the first 2 days of admission, since we wished to study nosocomial isolation
- inter-hospital transfers, since their total prior hospital stay was unknown.

### Statistical analysis

We used Poisson regression to estimate the incidence of nosocomial MRSA infection, including as denominator one day for every part of a calendar day spent in ORH hospitals >2 days after admission (providing the patient had not had an outcome within 2 days of this admission) up to the earliest of discharge, death or the MRSA outcome; and numerator whether or not MRSA was isolated from a sample taken on that day for each patient. Patients could contribute more than one nosocomial isolation to analysis if these occurred in different admissions. A succession of simple two trend models were fitted to explore changes in rate over calendar time, and the model minimising the Akaike Information Criterion (AIC[16]) presented, providing this two-slope model was a significantly better fit than a single trend model (similar results obtained using negative binomial regression allowing extra variability in monthly rates, and similar relationship between outcomes using three trend models). All analyses were unadjusted reflecting our objective of evaluating MRSA isolations as a surveillance measure. To investigate the ability to reliably detect similar trends to those we observed in ORH in other hospitals, we simulated genuine reductions in different event rates across two equal periods of observation of varying lengths, and related the probability of detecting this reduction as being statistically significant to common sizes of hopsital/division/speciality. Stata 9.2 was used for all analyses.

### ETHICS APPROVAL

Not required as linkage was anonymous; approval for the study obtained from the Caldicott guardian as with our previous studies.

## Results

Repeated isolation of MRSA from different clinical samples from a single patient is common; to estimate the number of infections, we determined the first clinical isolation (FCI) of MRSA for each patient admitted between 1 April 1998 and 1 July 2006, initially considering all clinical samples (excluding screens). 8% of FCIs >2 days after admission were from blood, with 15% from other normally sterile locations (including pus and periprosthetic samples), 10% from respiratory samples, 59% from surface cultures (e.g. ulcers and wounds), and the remaining 8% from urine specimens, a total of 3450 FCIs (Table 1). If MRSA isolations from surface cultures and urine samples (whose clinical significance can be difficult to assess in some individuals) are excluded, over the same period there were 1464 FCIs. If only blood cultures were included, there were 441 FCIs, with bacteraemia (positive blood culture) being the first nosocomial MRSA isolation during the admission in only 269 cases.

**Table 1:** Comparison of characteristics of first MRSA isolation from various clinical sites >2 days after admission to Oxford Hospitals, 1998–2006

		Bacteraemia N = 441	Blood, respiratory, sterile site N = 1464	All clinical isolates N = 3450	Global	(1) vs (2)	(1) vs (3)	(2) vs (3)
Factor	Subcategory	n (%) or median (IQR)	n (%) or median (IQR)	n (%) or median (IQR)	p*	p*	p*	p*
**Site** **	blood	441 (100%)	346 (24%)	269 (8%)	-	-	-	-
	sterile site		729 (50%)	527 (15%)				
	respiratory		389 (27%)	352 (10%)				
	surface/genital swab			2031 (59%)				
	urine			271 (8%)				
**Financial year of positive**	1998	20 (5%)	91 (6%)	204 (6%)	0.05	0.68	0.14	0.04
	1999	32 (8%)	115 (8%)	248 (7%)				
	2000	44 (10%)	153 (11%)	365 (11%)				
	2001	54 (13%)	196 (14%)	401 (12%)				
	2002	74 (18%)	248 (17%)	523 (16%)				
	2003	92 (22%)	258 (18%)	587 (18%)				
	2004	59 (14%)	187 (13%)	526 (16%)				
	2005	45 (11%)	170 (12%)	489 (15%)				
	2006†	21	46	107				
**Days from admission to positive**		16 (7–31)	14 (7–28)	15 (8–30)	0.04	0.07	0.67	0.02
**Sex**	female	167 (38%)	569 (39%)	1471 (43%)	0.02	0.71	0.06	0.01
**Age at admission**	(years)	73 (63–81)	72 (58–80)	74 (60–82)	0.0003	0.05	0.59	0.0001
**Any previous MRSA** ‡	screening or clinical sample	55 (12%)	275 (19%)	684 (20%)	0.001	0.002	<0.0001	0.40
	*screening sample only*	*16 (4%)*	*39 (3%)*	*82 (2%)*				
	*clinical sample only*	*18 (4%)*	*106 (7%)*	*266 (8%)*				
	*clinical and screen*	*21 (5%)*	*130 (9%)*	*336 (10%)*				
**Never previously admitted to ORH**		120 (27%)	401 (27%)	861 (25%)	0.16	0.94	0.30	0.07
**Last discharge from ORH (days before this admission)**		56 (15–290)	59 (17–272)	69 (18–314)	0.22	0.66	0.20	0.16
**Number of prior ORH admissions**		2 (0–4)	2 (0–4)	2 (1–4)	0.04	0.62	0.06	0.04
**Days in ORH prior to this admission**		10 (0–33)	11 (0–37)	13 (1–41)	0.02	0.37	0.03	0.04
**Admission speciality**	trauma/A&E/ortho/cardio	34 (8%)	172 (12%)	352 (10%)	0.002	0.004	0.006	0.05
	specialityobs/gynae/paeds/ENT	16 (4%)	88 (6%)	219 (6%)				
	haemat/onc/nephr	29 (7%)	50 (3%)	145 (4%)				
	surgery	162 (37%)	511 (35%)	1086 (31%)				
	medicine: elective	5 (1%)	20 (1%)	55 (2%)				
	medicine: emergency	195 (44%)	623 (43%)	1593 (46%)				
**Non-medical emergency**		145 (33%)	489 (33%)	1043 (30%)	0.07	0.84	0.25	0.03

* univariable p values from chi-squared or Kruskal-Wallis/ranksum tests for categorical and continuous variables respectively.

** first MRSA isolation from specified samples this admission (predominant site according to order above when MRSA isolated from multiple types of specimens on the same day).

† 3 months April to June 2006: percentages are of complete financial years only.

‡ any sample tested at ORH from 1 January 1995 onwards, during or outside of an ORH admission, but strictly before the current admission

Note:excluding repeated positive isolations within a single admission.

Notably, the characteristics of patients with bacteraemia, blood/respiratory/sterile site and any clinical MRSA isolation had many similarities (Table 1). For example, median ages were similar (73, 72 and 74 years respectively), as were the durations that the patients had been in hospital prior to the current isolation (median 16, 14 and 15 days respectively). While large numbers of non-bacteraemic isolations provide enough statistical power to show these differences may not be due to chance alone (Table 1), they are unlikely to be important clinically. The main difference was between the proportions of patients who had had MRSA isolated from any sample, clinical or MRSA screen, taken in or outside of hospital before this admission (12% for bacteraemia, 19% for blood/respiratory/sterile site isolation, and 20% for any clinical isolation, p = 0.001). There were also small differences in inpatient stay prior to the current admission (median 10, 11 and 13 days respectively, p = 0.02). We also observed the expected small variations by admission speciality (p = 0.002), with proportionately more bacteraemias in line-intensive specialities of haematology, oncology, and nephrology.

**Figure 1:**
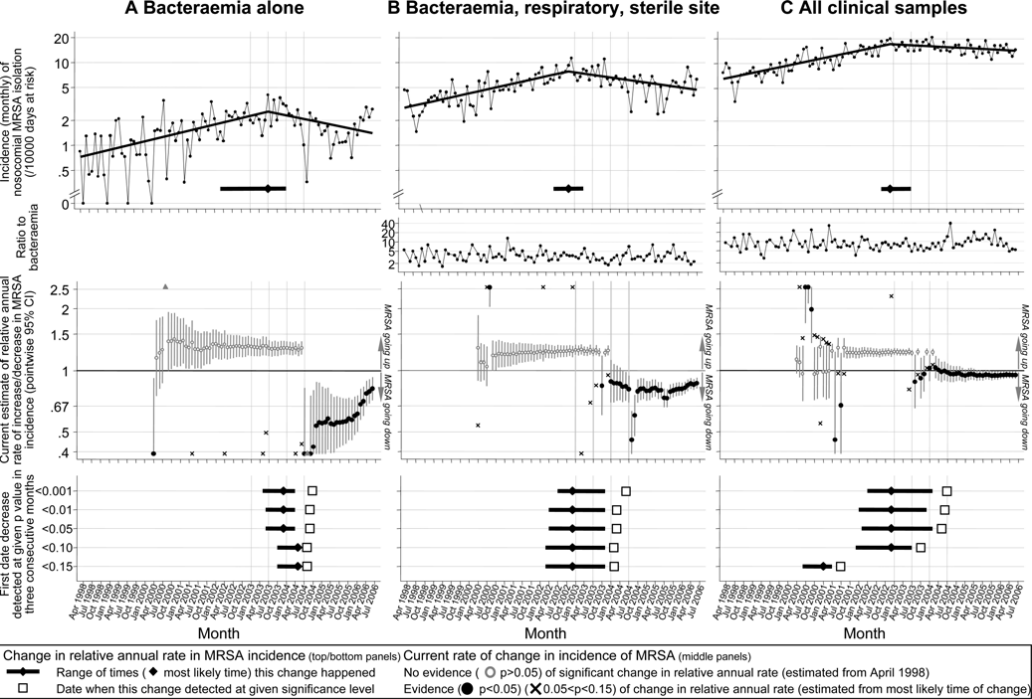
MRSA INCIDENCE OVER TIME. TOP PANELS: Monthly nosocomial MRSA incidence, and change in relative rates from blood cultures, the combination of bacteraemia, respiratory and sterile site samples, and all clinical samples. MIDDLE PANELS: estimates of relative annual rate of increase or decrease in incidence, calculated each month from April 2000 to June 2006. LOWER PANELS: the point at which changes in rate were most likely to have occurred, and when they could have been confidently detected by an infection control team monitoring trends.

Figure 1 (top panels) shows the monthly nosocomial MRSA incidence per 10,000 patient-days in ORH over calendar time. Inspection suggests that incidence of both MRSA bacteraemia and MRSA isolation from other clinical samples increased then declined, while their ratio changed little.

Poisson regression was used to assess the significance of these trends. We found that incidence of nosocomial MRSA bacteraemia increased by 27% per year to July 2003 (p<0.001, Table 2, solid line Figure 1 top panels) before declining by 18% per year thereafter (p = 0.002). Nosocomial MRSA bacteraemia/respiratory/sterile site and all clinical isolations similarly increased significantly (by 25% and 23% per year to November/December 2002 respectively) and then declined significantly (by 13% and 5% per year respectively) (all p<0.01), although their incidence was a median 3.3 and 8.0-fold higher than bacteraemia alone (Figure 1 second row of panels). While MRSA isolation declined significantly for all outcomes, using a multivariate Poisson model, estimates of the decline in all clinical MRSA isolations were slightly smaller than declines in bacteraemia (13% smaller per year [95% CI 1–26% smaller] p = 0.02) but more similar to declines in blood/respiratory/sterile site (4% smaller per year [15% smaller-5% larger] p = 0.39) (with similar results estimating declines after December 2002 for all outcomes). Analysing ratios of the incidence of bacteraemia/respiratory/sterile site or all clinical isolations to bacteraemia, we found no significant time trends. Similar results were obtained excluding patients with any MRSA positive clinical or screening sample before the current admission (leaving 386 bacteraemias, 1189 bacteraemia/respiratory/sterile site and 2776 clinical samples in total), although the most likely time for the reduction in MRSA bacteraemias to have started was slightly earlier (December 2002 compared with July 2003 in all patients). Similar results were also obtained comparing non-overlapping groups of bacteraemic versus non-bacteraemic MRSA isolations. In summary, multiple methods of analysis suggest that changes in incidence of first clinical MRSA isolation are similar whether blood cultures, all clinical samples, or a subset of all clinical samples are studied.

**Table 2:** Changes in rates of first MRSA isolation >2 days after admission to Oxford Hospitals from various clinical sites over 1998–2006

	Bacteraemia	Blood, respiratory, sterile site	All clinical isolates
Positive isolations	441	1464	3450
- mean isolations per month in 1999	2.3	9.4	19.4
- mean isolations per month in 2002	5.8	20.8	42.7
- mean isolations per month in 2005	3.5	13.2	40.6
Patient days at risk from >2 days after admission to the earliest of discharge, death or MRSA isolation	2,676,180	2,654,119	2,617,870
Most likely time that trend in rates changes (“changepoint”)*	July 2003	November 2002	December 2002
Fold change in isolation rate per year to this changepoint (HR (95% CI) p)	1.27 _(1.18–1.37)_<0.001	1.25 _(1.19–1.31)_<0.001	1.23 _(1.19–1.27)_<0.001
Fold change in isolation rate per year subsequently (HR (95% CI) p)	0.82 _(0.72–0.93)_ 0.002	0.87 _(0.82–0.92)_<0.001	0.95 _(0.92–0.98)_ 0.008
*Heterogeneity p value*	*het p<0.001*	*het p<0.001*	*het p<0.001*
Range of times of rate trend change which cannot be distinguished statistically from this changepoint**	March 2002 to January 2004 (22 months)	June 2002 to April 2003 (10 months)	September 2002 to July 2003 (10 months)
First month after April 2000 when data up to and including this month suggest a date in this range is the most likely time for a change in rate trend with p<0.05	July 2004	October 2003	August 2003
*(p, date identified as most likely change)*	*(p = 0.005, Jan 2004)*	*(p = 0.03, Dec 2002)*	*(p = 0.03, Dec 2002)*

* identified as per Methods (see Figure 1).

** based on difference in AIC of <3.84 from the best-fitting changepoint model.

Given recent intensive infection control initiatives, a key question is when the increase in MRSA incidence actually reversed. Studying our hospital Trust from 1998–2006, the most likely time that the reduction in MRSA incidence started varied a little with outcome (July 2003, November 2002 and December 2002 respectively), but the range of times that could not be distinguished statistically from this most likely time overlapped substantially (Table 2, Figure 1 top panels). This illustrates the difficulty in determining exactly when changes in incidence have occurred given natural random (stochastic) variation, even in large Trusts. Notably, there was much greater uncertainty in when the change in MRSA bacteraemia might have occurred (22 months from March 2002 to January 2004), compared to other outcomes (10 months).

Continuous review and monthly reporting of MRSA bacteraemia is a mandatory requirement in the UK. We considered to what extent the three MRSA outcome measures described above (bacteraemia, isolation from blood/respiratory/sterile sites, and all clinical isolates) could help an infection control team decide whether and how MRSA incidence was changing, by plotting for data up to and including each month from April 2000 through June 2006, the best current estimate of the relative annual rate of increase/decrease in MRSA incidence classified by the statistical evidence for this having changed (Figure 1, middle panels). To mid-2001 there was weak evidence that MRSA incidence was going up for each outcome. From mid-2001 to mid-2003, it was clear that MRSA incidence from all clinical and blood/respiratory/sterile site samples was increasing significantly, but the evidence for increasing incidence of nosocomial MRSA bacteraemia much less strong (particularly in 2002). Through the second-half of 2003 it became clear that MRSA incidence from all clinical and blood/respiratory/sterile site samples was now declining significantly compared to their earlier increase. From mid-2004 onwards, there was also statistical evidence for a decline in MRSA bacteraemias, but the confidence intervals around the estimated reduction were far wider, and drift upwards, with a short period (July/August 2004) with only 3 and 1 nosocomial bacteraemias respectively having large influence. Considering when the increases in MRSA incidence were first consistently estimated to have reversed (Figure 1, bottom panels), we found that the decline in incidence of all clinical nosocomial MRSA from December 2002 was identified at the 10% significance level in October 2003, compared to February 2004 for blood/respiratory/sterile site samples. Changes with greater statistical certainty (p<0.1) and in bacteraemias took longer to detect. In summary, throughout the period studied, use of blood culture alone as a measure afforded more variable estimates of current trend than other measures, and detected changes in incidence later.

**Figure 2:**
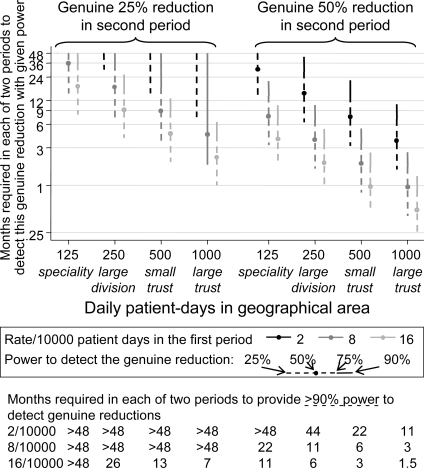
TIME REQUIRED TO DETECT A CHANGE IN MRSA INCIDENCE. Durations of two equal periods required to detect a 25% (left panel) or 50% (right panel) reduction in rate of MRSA isolation, for a variety of sizes of hospital unit.

Oxford Radcliffe Trust is one of the largest Acute Hospital Trusts in the UK. An average Acute Hospital Trust is about half the size, and a small Trust on the 25th centile of the UK distribution is about 35% the size[2]. We therefore considered how long a period of observation would be needed to reliably (>90% probability/power) detect the required 50% or a more modest 25% reduction in MRSA incidence in varying sizes of hospital populations, from a baseline incidence similar to that we observed for bacteraemias (2/10000 patient days), bacteraemia/respiratory/sterile site (8/10000 patient days) and all clinical specimens (16/10000 patient days), under a simple Poisson assumption (Figure 2). With the least common outcome (blood cultures), periods of ∼12 months were needed even in the largest hospitals to reliably detect 50% reductions, and periods of 2 or more years were needed to provide the same power in smaller clinical units. In contrast, with the most common outcome (all clinical samples), 50% reductions could be reliably detected in ∼12 months even in areas the size of speciality groups, and smaller changes of 25% could be detected in 6–12 months in larger hospitals.

## Discussion

Here we show that whilst trends over time in nosocomial bacteraemic and non-bacteraemic isolates of MRSA are similar in a large UK Acute Hospital Trust over a 10 year period, non-bacteraemic clinical isolates are about 8-fold more frequent, with their ratio relatively constant. For reasons which are not investigated here, incidence of both outcomes declined progressively from early 2003, offering the opportunity to assess the ability of isolations from different types of clinical specimen to monitor changes in MRSA. Similar patient characteristics and time trends suggest that similar populations are likely to be at risk for both bacteraemic and non-bacteraemic isolation, with bacteraemia an indicator of the most severe infection across a spectrum rather than having different epidemiology. Indeed, there is extensive overlap between these groups, with two thirds of patients with bacteraemia also having MRSA isolated from a non-blood source during the same admission, around half of these isolations being prior to the bacteraemia (rather than prompted by a positive blood culture). Due to the vastly different incidence between bacteraemia and non-bacteraemic isolation, all clinical isolations was a more sensitive measure for detecting changes in MRSA rates in our Trust over 1998–2006 than bacteraemia alone.

What are the limitations of the proposed surveillance measure, given that, as is likely, practical issues of data collection and computation can be readily addressed by appropriate information technology? Firstly, as we focussed on potentially nosocomially acquired infections, we did not study patients in the first two days of admission, a group we have previously shown to account for about 25% of all hospital bacteraemia in the UK[17]. Secondly, we measured isolation from diverse specimen types, including urine, wound and respiratory cultures. Compared with blood stream isolates, individually, the significance of these may be more difficult to judge. In many cases, given the clinical context and high prevalence of MRSA as the cause of many nosocomial infection syndromes, it is likely to reflect a nosocomial infection with significant morbidity, mortality and cost[10]–[15]. In other situations, the isolation may reflect colonisation, but given that colonisation itself is associated with substantial risk of subsequent infection[7]–[9] and that there has been a clinical indication for taking a specimen (as opposed to a screen of an otherwise healthy appearing individual), its inclusion in a surveillance measure may be justified. All clinical MRSA isolations may be a good “surrogate” for more severe bacteraemia, with a substantial increase in power which makes comparisons across fair smaller geographical areas feasible.

An additional caveat is that by necessity such a surveillance measure is restricted to clinical samples which happened to be taken, and sampling frequency given particular conditions (such as surgical wounds) may be determined not only by the clinical condition but also by other factors (e.g. local policies). However, this is probably also true of blood cultures, since indications for blood culture are controversial in many clinical settings. We believe these issues are largely irrelevant provided one has a goal of determining success of infection control within an institution, if the sampling indications remain similar over time, which is an assumption behind much passive infectious disease surveillance. Indeed, because of the increased frequency of our outcome measure, we suggest our proposed measure has many advantages over the inherently variable and infrequent bacteraemia based outcome currently used[5].

Information provision to relevant individuals forms part of universally recognised definitions of effective surveillance[18]. If, as is clear, MRSA control requires action at hospital or departmental level, “information for action” should be available at these levels[18]. Our proposed measure offers an improvement on the current situation, where the basis on which hospital-wide actions are intensified are cross-institutional rankings heavily determined by stochastic variation[5] and should be validated in other hospitals.
